# A Molecular Switch Abrogates Glycoprotein 100 (gp100) T-cell Receptor (TCR) Targeting of a Human Melanoma Antigen*

**DOI:** 10.1074/jbc.M115.707414

**Published:** 2016-02-25

**Authors:** Valentina Bianchi, Anna Bulek, Anna Fuller, Angharad Lloyd, Meriem Attaf, Pierre J. Rizkallah, Garry Dolton, Andrew K. Sewell, David K. Cole

**Affiliations:** From the Division of Infection and Immunity and Systems Immunity Research Institute, Cardiff University School of Medicine, Heath Park, Cardiff CF14 4XN, United Kingdom

**Keywords:** cancer, melanoma, surface plasmon resonance (SPR), T-cell receptor (TCR), X-ray crystallography, CD8+ T-cells, gp100, heteroclitic peptides, peptide human leukocyte antigen (pHLA)

## Abstract

Human CD8^+^ cytotoxic T lymphocytes can mediate tumor regression in melanoma through the specific recognition of HLA-restricted peptides. Because of the relatively weak affinity of most anti-cancer T-cell receptors (TCRs), there is growing emphasis on immunizing melanoma patients with altered peptide ligands in order to induce strong anti-tumor immunity capable of breaking tolerance toward these self-antigens. However, previous studies have shown that these immunogenic designer peptides are not always effective. The melanocyte differentiation protein, glycoprotein 100 (gp100), encodes a naturally processed epitope that is an attractive target for melanoma immunotherapies, in particular peptide-based vaccines. Previous studies have shown that substitutions at peptide residue Glu^3^ have a broad negative impact on polyclonal T-cell responses. Here, we describe the first atomic structure of a natural cognate TCR in complex with this gp100 epitope and highlight the relatively high affinity of the interaction. Alanine scan mutagenesis performed across the gp100^280–288^ peptide showed that Glu^3^ was critically important for TCR binding. Unexpectedly, structural analysis demonstrated that the Glu^3^ → Ala substitution resulted in a molecular switch that was transmitted to adjacent residues, abrogating TCR binding and T-cell recognition. These findings help to clarify the mechanism of T-cell recognition of gp100 during melanoma responses and could direct the development of altered peptides for vaccination.

## Introduction

Cytotoxic T-cells can mediate a specific response against autologous melanoma cells by recognizing tumor-derived peptides presented at the cell surface by human leukocyte antigen (pHLA).^6^ In particular, epitopes encoded by differentiation melanocyte proteins may represent shared melanoma-associated antigens targeted by T-cell receptors (TCRs) on patients' lymphocytes (1). Glycoprotein 100 (gp100) has been a widely studied target for melanoma immunotherapy. This 661-amino acid long melanoma differentiation antigen is a melanosome matrix protein involved in melanosome maturation and melanin synthesis (2). *In vivo*, the protein has significantly differential expression between tumor cells, being often overexpressed in all stages of melanoma progression, compared with normal melanocytes (3).

Previous studies showed that gp100 encoded epitopes are recognized by tumor-infiltrating lymphocytes and circulating T-cells, associated with tumor regression in metastatic melanoma patients after adoptive therapy (4–7). Among these, the nonamer epitope gp100^280–288^ (YLEPGPVTA) was originally shown to be recognized by HLA-A*0201^+^ tumor-infiltrating lymphocytes from melanoma patients (8) and subsequently eluted from HLA-A*0201 molecules on melanoma cells (9). Immunization with gp100^280–288^ peptide has been shown to stimulate an *in vitro* polyclonal T-cell response in the context of HLA-A*0201, present in 49% of Caucasian individuals (10). These findings generated renewed interest in developing gp100-based anti-melanoma vaccines. However, we and others have previously shown, through direct biophysical measurements, that anti-cancer TCRs bind to their cognate pHLA with affinities that are approximately 1 order of magnitude weaker than those of pathogen-specific TCRs (11, 12). Thus, altered peptide ligands, with improved primary HLA anchor residues (heteroclitic peptides), have been designed for a few melanoma-associated antigens in order to increase immunogenicity (6, 10, 13). Among these, the heteroclitic version of gp100^280–288^ (in which a valine replaces alanine at anchor position 9 to improve pHLA stability (14)) enhanced the induction of melanoma-reactive cytotoxic T lymphocytes *in vitro* and has been successfully used in clinical trials (15). Another heteroclitic form of gp100^280–288^, in which peptide residue Glu^3^ was substituted to Ala, abrogated recognition by a polyclonal population of gp100^280–288^-specific T-cells (16, 17). Thus, a more complete understanding of the molecular mechanisms underlying gp100^280–288^ targeting by specific TCRs is needed to direct the design of improved altered peptide ligands.

Previous studies using another HLA-A*0201-restricted melanoma-derived epitope have demonstrated that even minor changes in peptide anchor residues can substantially alter T-cell recognition in unpredictable ways (13, 18). In order to aid in the future design of enhanced peptide vaccines based on gp100^280–288^, we solved the ternary atomic structure of a human TCR in complex with the heteroclitic gp100^280–288^ peptide. We then used a peptide scanning approach to demonstrate the impact of peptide substitutions on TCRs from two different T-cell clones by performing in depth biophysical and functional experiments. These data demonstrate that modification of peptide residues outside of the TCR binding motif can have unpredictable knock-on effects (a modification to a residue that affects an adjacent residue indirectly) on adjacent peptide residues that abrogate TCR binding and T-cell recognition. Indeed, even conservative peptide substitutions can have unexpected consequences for T-cell recognition due to knock-on structural changes in the HLA-bound peptide. Our findings provide a molecular explanation for the sensitivity to substitutions at gp100^280–288^ peptide residue Glu^3^ (16, 17) and represent the first example of the structural mechanisms underlying T-cell recognition of this important therapeutic target for melanoma.

## Experimental Procedures

### 

#### 

##### Generation of Expression Plasmids

The PMEL17 TCR (TRAV21 TRBV7-3) (12) and gp100 TCR (TRAV17 TRBV19) (11) are both specific for the human HLA-A*0201 restricted YLE epitope (gp100^280–288^, sequence YLEPGPVTA). For each TCR, a disulfide-linked construct was used to produce the soluble α- and β-chain domains (variable and constant) (19, 20). The HLA*0201 α-chain and β2m sequences were generated by PCR cloning. All sequences were confirmed by automated DNA sequencing (Cardiff Biotechnology Service). For surface plasmon resonance (SPR) experiments, the soluble HLA-A*0201 α-chain was tagged with a biotinylation sequence, as described previously (21). All four constructs (TCRα, TCRβ, HLA-A*0201 α-chain, and β2m) were inserted into separate pGMT7 expression plasmids under the control of the T7 promoter (20).

##### Protein Expression, Refolding, and Purification

Competent Rosetta DE3 *Escherichia coli* cells were used to produce the PMEL17 TCR, gp100 TCR α and β chains, and HLA-A*0201 α and β_2_m chains in the form of inclusion bodies, using 0.5 m isopropyl 1-thio-β-d-galactopyranoside to induce expression. Soluble PMEL17 TCR, gp100 TCR, and pHLA proteins were refolded as described previously (19, 20), purified by anion exchange (Poros 50HQ, Life Technologies, Cheshire, UK) and size exclusion chromatography (S200 GR, GE Healthcare, Buckinghamshire, UK). For the pHLAs, HLA-A*0201 was refolded with YLEPGPVTA (A2-YLE), YLEPGPVT**V** (A2-YLE-9V), **A**LEPGPVTA (A2-YLE-1A), YL**A**PGPVTA (A2-YLE-3A), YLE**A**GPVTA (A2-YLE-4A), YLEP**A**PVTA (A2-YLE-5A), YLEPG**A**VTA (A2-YLE-6A), YLEPGP**A**TA (A2-YLE-7A), or YLEPGPV**A**A (A2-YLE-8A).

##### SPR Analysis

The binding analysis was performed using a BIAcore 3000 or Biacore T100 equipped with a CM5 sensor chip as reported previously (21). Briefly, 500–600 response units of biotinylated pHLA-I complexes were immobilized to streptavidin, which was chemically linked by amine coupling to the chip surface. Biotinylated pHLA-I complexes were prepared as described previously (21) and injected at a slow flow rate (10 μl/min) to ensure a uniform distribution on the chip surface. Results were analyzed using BIAevaluation^TM^ version 3.1, Microsoft Excel^TM^, and Origin^TM^ version 6.0. For equilibrium analysis, 9–10 serial dilutions of concentrated TCR were injected over the relevant sensor chip. The equilibrium-binding constant (*K_D_*(*E*)) values were calculated using a nonlinear curve fit (*y* = (P1*x*)/(P2 + *x*)). For the thermodynamics experiments, *K_D_* values determined by SPR at different temperatures were used with the standard thermodynamic equationΔ*G*^0^ = −*RT*ln*K_D_*. The thermodynamic parameters were calculated according to the Gibbs-Helmholtz equation (Δ*G*^0^ = Δ*H* - *T*Δ*S*^0^). The binding free energies, Δ*G*^0^ (Δ*G*^0^ = −*RT*ln*K_D_*), were plotted against temperature (*K*) using nonlinear regression to fit the three-parameter equation, (*y* = Δ*H* + ΔCp*(*x* − 298) − x*Δ*S* − x*ΔCp*ln(*x*/298)). For kinetics analysis, the *K*_on_ and *K*_off_ values were calculated assuming 1:1 Langmuir binding, and the data were analyzed using a global fit algorithm (BIAevaluation^TM^ version 3.1). All SPR experiments were conducted in triplicate.

##### Crystallization, Diffraction Data Collection, and Model Refinement

All protein crystals were grown at 18 °C by vapor diffusion via the sitting drop technique. 200 nl of 1:1 molar ratio TCR and pHLA-I (10 mg/ml) in crystallization buffer (10 mm Tris, pH 8.1, and 10 mm NaCl) was added to 200 nl of reservoir solution. PMEL17 TCR·A2-YLE-9V crystals were grown in 0.2 m sodium sulfate, 0.1 m Bistris propane, pH 6.5, 20% (w/v) PEG 3350. Crystals of pHLA complexes were grown at 18 °C by seeding into hanging drops of 0.5 μl of seeding solution + 1 μl of complex + 1 μl of 0.1 m Hepes, pH 7.5, 0.2 m ammonium sulfate, 25% PEG 4000 (22). Data were collected at 100 K at the Diamond Light Source (Oxfordshire, UK). All data sets were collected at a wavelength of 0.976 Å using an ADSC Q315 CCD detector. Reflection intensities were estimated with the XIA2 package (23), and the data were scaled, reduced, and analyzed with SCALA and the CCP4 package (24). Structures were solved by molecular replacement using PHASER (25). Sequences were adjusted with COOT (26) and the models refined with REFMAC5 (27). Graphical representations were prepared with PyMOL (28). Data reduction and refinement statistics are shown in Table 1. The reflection data and final model coordinates were deposited in the Protein Data Bank (entries 5EU6 (PMEL17 TCR·A2-YLE-9V), 5EU3 (A2-YLE), 5EU4 (A2-YLE-3A), and 5EU5 (A2-YLE-5A)).

##### Isothermal Titration Calorimetry (ITC)

ITC experiments were performed using a Microcal VP-ITC (GE Healthcare) as described previously (29), with 30 μm pHLA-I in the calorimeter cell and 210 μm soluble PMEL17 TCR in the syringe. Buffer conditions were 20 mm Hepes (pH 7.4) containing 150 mm NaCl, and 20 injections of 2 μl each were performed. Results were processed and integrated with the Origin^TM^ version 6.0 software distributed with the instrument. ITC experiments were performed in duplicate.

##### Lentivirus Generation and Transduction of CD8^+^ T-cells

Lentivirus particles were generated by combining packaging plasmids pRSV, pMDL, and pVSG-V with a lentivirus plasmid bearing the gp100 TCR construct (provided by Immunocore Ltd., Oxford, UK) and used to CaCl_2_-transfect HEK293T/17 (ATCC) cells. Supernatant was collected after 24- and 48-h incubations, and lentiviral stocks were concentrated by ultracentrifugation. Primary CD8^+^ T-cells were obtained by standard density gradient centrifugation from donor blood bags, followed by positive selection using CD8 microbeads (Miltenyi Biotec). Cells were activated overnight with anti-CD3/CD28 Dynabeads (Invitrogen) (1:1) and transduced with concentrated lentivirus particles. Transduction efficiency was determined after 72 h by flow cytometry after staining with the relevant anti-TCRVβ mAb (BD Biosciences). Untransduced cells or MEL5 TCR (specific for the Melan-A/MART-1 epitope ELAGIGILTV)-transduced cells were used as controls (data not shown). Transductions were performed using primary CD8^+^ T-cells from three different anonymous donors.

##### Measurement of MIP-1β and TNFα by ELISA

To quantify the production of MIP-1β and TNFα, 6 × 10^4^ T2 target cells were pulsed with peptide as indicated for 1 h and added to 3 × 10^4^ overnight rested T-cells. Following overnight incubation, cells were pelleted, and the culture supernatant was harvested for measurement of MIP-1β and TNFα by ELISA according to the manufacturer's protocol (R&D Systems). Each data point represents the average of duplicate measurements.

##### Cytotoxicity Assay

Cytotoxic assays in this study were performed in a standard 4-h ^51^Cr release assay. Briefly, 2 × 10^3^ T2 cells were labeled with ^51^Cr (PerkinElmer Life Sciences) and then pulsed with peptide at the indicated concentration and used as target cells. Effector and target cells, at an effector/target ratio of 5, were incubated for 4 h at 37 °C, and the supernatant was harvested. Target cells were also incubated alone or with 5% Triton X-100 detergent to give the spontaneous and total ^51^Cr released from the target cells, respectively. ^51^Cr release was determined by γ-counting (1450 Microbeta counter, PerkinElmer Life Sciences). The percentage of specific cell lysis was calculated according to the following formula: (experimental release (with effector and target cells) − spontaneous release from target cells)/(total release from target cells − spontaneous release from target cells) × 100. Each data point represents the average of duplicate measurements.

## Results

### 

#### 

##### Two Distinct Anti-gp100 TCRs Share Similar Binding Hot Spots

The CD8^+^ T-cell responses directed against gp100^280–288^have been shown to be polyclonal in nature (16, 17). Along with the two TCRs under investigation here, the sequences of a further two TCRs have been published, demonstrating diverse gene usage and different CDR3 loop sequences (Table 1). Despite these differences, previous studies of T-cell responses to gp100^280–288^ have demonstrated that modifications to peptide residue Glu^3^ can broadly effect activation of gp100^280–288^-specific T-cells (16, 17). Thus, in order to study the individual contribution of the peptide residues involved in TCR recognition of gp100^280–288^, particularly in relation to peptide residues Glu^3^, an alanine scan mutagenesis was performed across the peptide backbone, and TCR binding affinity was evaluated by SPR experiments. Residues P2 and P9, which are known to be important for HLA-A*0201 binding (30) were not assessed; in addition, the P9 residue was an Ala in the native sequence. The heteroclitic YLE-9V peptide, which has been shown to be a better agonist than the wild type sequence (10), was included in the experiment. SPR experiments were conducted with two distinct gp100^280–288^-specific TCRs: PMEL17 TCR (*TRAV21 TRBV7-3*) and gp100 TCR (*TRAV17 TRBV19*). PMEL17 TCR bound both A2-YLE and A2-YLE-9V with similar affinities (*K_D_* = 7.6 and 6.3 μm, respectively), consistent with the fact that the YLE-9V variant was originally designed in such a way as to increase peptide-HLA binding affinity without significantly altering TCR recognition of the pHLA complex (10) (Table 2). The gp100 TCR demonstrated a similar pattern, although at weaker affinities, of *K_D_* = 26.5 and 21.9 μm, for A2-YLE and A2-YLE-9V, respectively. With the exception of A2-YLE-3A and A2-YLE-5A, both the PMEL17 and gp100 TCRs tolerated substitutions in the native gp100^280–288^ peptide, albeit with reduced binding affinity, although substitutions at the peptide C terminus generally reduced binding affinity to a greater extent than at the N terminus. Substitution of peptide residue 5 to alanine reduced the affinity for both TCRs to *K_D_* >1 mm. Interestingly, replacement of Glu by Ala in position 3 completely abrogated binding by PMEL17 and gp100 TCRs, suggesting that the Glu at p3 was a dominant contact for both TCRs. Our results are supported by a recent study of gp100^280–288^altered peptide ligands, which described YLE-3A as a null agonist for a different TCR (17). Our data indicated that both PMEL17 and gp100 TCRs used a similar overall binding mode, focused around peptide residues 3 and 5 with supporting interactions toward the N terminus of the peptide. In combination with other data in this system (17), alanine substitution data suggest that disparate TCRs adopt a similar binding mode on A2-YLE, where position 3 dominates recognition. In order to confirm this hypothesis, we crystallized the PMEL17 TCR in complex with A2-YLE-9V.

**TABLE 1:** **Alignment of TCR CDR3 regions of PMEL17, gp100, MPD (16), and 296 gp100-specific TCRs (16)**

TCR	CDR1α	CDR2α	CDR3α	CDR1β	CDR1β	CDR1β
PMEL17	DSAIYN	IQSSQRE	CAVLSSGGSNYKLTFG	SGHTA	FQGTGA	CASSFIGGTDTQYFG
gp100	TSINN	IRSNERE	CATDGDTPLVFG	LNHDA	SQIVND	CASSIGGPYEQYFG
MPD	KALYS	LLKGGEQ	CGTETNTGNQFYFG	SGHDY	FNNNVP	CASSLGRYNEQFFG
296	DSASNY	IRSNVGE	CAASTSGGTSYGKLTFG	MNHEY	SMNVEV	CASSLGSSYEQYFG

**TABLE 2:** **Affinity analysis (*K_D_*) of PMEL17 TCR and gp100 TCR to gp100^280–288^ peptide variants**

Peptide sequence	Peptide	PMEL17 TCR (*TRAV21 TRBV7-3*), affinity *K_D_*	gp100 TCR (*TRAV17 TRBV19*), affinity *K_D_*
YLEPGPVTA	YLE	7.6 ± 2 μm	26.5 ± 2.3 μm
YLEPGPVTV	YLE-9V	6.3 ± 1.2 μm	21.9 ± 2.4 μm
ALEPGPVTA	YLE-1A	15.9 ± 4.1 μm	60.6 ± 5.4 μm
YLAPGPVTA	YLE-3A	No binding	No binding
YLEAGPVTA	YLE-4A	19.7 ± 1.3 μm	144.1 ± 7.8 μm
YLEPAPVTA	YLE-5A	>1 mm	>1 mm
YLEPGAVTA	YLE-6A	11.4 ± 2.7 μm	954.9 ± 97.8 μm
YLEPGPATA	YLE-7A	31.1 ± 4 μm	102.0 ± 9.2 μm
YLEPGPVAA	YLE-8A	38.1 ± 7.4 μm	121.0 ± 7.5 μm

##### The PMEL17 TCR Utilizes a Peptide-centric Binding Mode to Engage A2-YLE-9V

**FIGURE 1.**
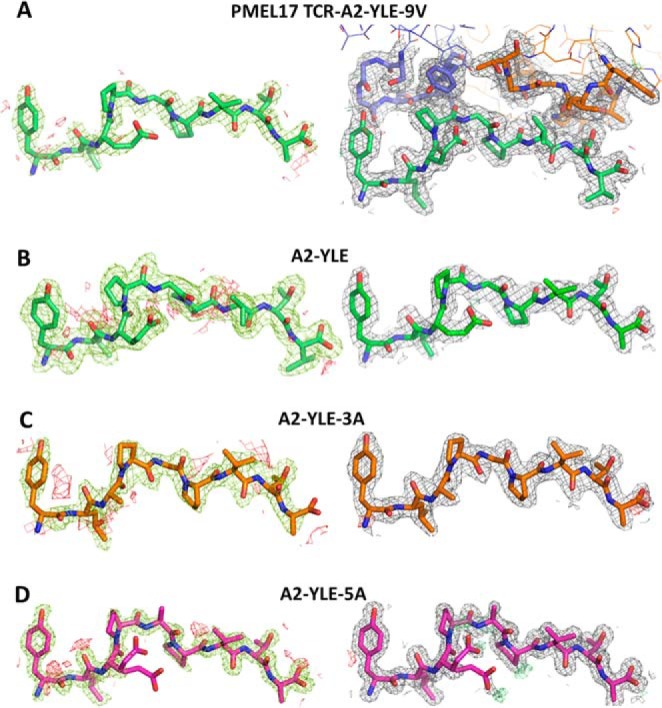
**Density plot analysis.** The *left column* shows omit maps in which the model was refined in the absence of the peptide. Difference density is contoured at 3.0 σ; positive contours are shown in *green*, and negative contours are *red*. The *right-hand column* shows the observed map at 1.0 σ (shown as *gray mesh around stick representations* of the protein chains) after subsequent refinement using automatic non-crystallographic symmetry restraints applied by REFMAC5. *A*, model for PMEL17 TCR·A2-YLE-9V with the TCR CDR3 loops *colored blue* (α chain) and *orange* (β chain) and the peptide in *green*; *B*, model for A2-YLE with the peptide *colored dark green*; *C*, model for A2-YLE-3A with the peptide *colored orange* (for A2-YLE-3A, there were two molecules in the asymmetric unit, but these were virtually identical in terms of omit and density maps, so only copy 1 is shown here); *D*, model for A2-YLE-5A with the peptide *colored pink*.

**FIGURE 2.**
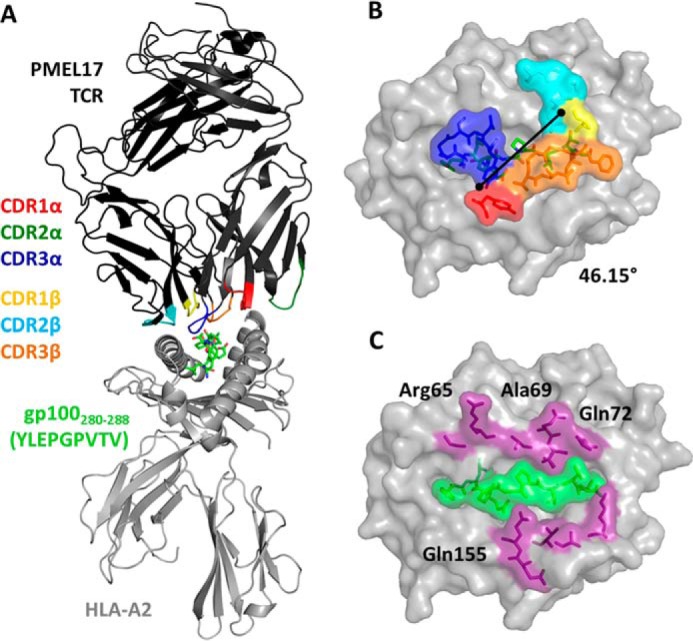
**Overview of the PMEL17 TCR in complex with A2-YLE-9V.** *A*, schematic representation of the PMEL17 TCR·A2-YLE-9V complex. The TCR α-chain and TCR β-chain are *dark gray* and *black*, respectively; TCR CDR loops are shown (*red*, CDR1α; *dark green*, CDR2α; *blue*, CDR3α; *yellow*, CDR1β; *aqua*, CDR2β; *orange*, CDR3β); the HLA-A*0201 is depicted in *gray*. The YLE-9V peptide is represented in *green sticks. B*, *surface* and *stick representation* of residues of the PMEL17 TCR CDR loops (*color-coded* as in *A*) that contact the A2-YLE surface (A2, *gray*; YLE-9V, *green sticks*). *Black diagonal line*, crossing angle of the TCR with respect to the long axis of the YLEPGPVTV peptide (46.15°). *C*, contact footprint of the PMEL17 TCR on the A2-YLE-9V surface (A2, *gray*); *purple* and *green* (*surface* and *sticks*) indicate HLA-A*0201 and YLE residues, respectively, contacted by the gp100 TCR (cut-off of 3.4 Å for hydrogen bonds and 4 Å for van der Waals contacts).

To understand why TCR recognition of gp100^280–288^ was highly sensitive to some of the substitutions in the native peptide sequence, we determined the crystal structure of the PMEL17 TCR in complex with A2-YLE-9V at 2.02 Å resolution with crystallographic *R*_work_/*R*_free_ ratios within accepted limits (Table 3) as shown in the theoretically expected distribution (31). Electron density around the TCR·pHLA contact interface was unambiguous (Fig. 1). The PMEL17 TCR was centrally positioned over the exposed residues of the peptide (Fig. 2, *A* and *B*) and used a conventional diagonal orientation (crossing angle = 46.15°, calculated as in Ref. 32), with the α-chain positioned over the α2 helix of the HLA-I binding groove and the β-chain over the α1 helix. All but the CDR2α loop were involved in contacting A2-YLE-9V, with the CDR3α and CDR3β “sitting” on the central axis of the antigen-binding cleft, making contacts with both the peptide and α-helices of the HLA (Fig. 1*B*). The PMEL17 TCR made approximately the same number of peptide-mediated contacts and HLA-A*0201 interactions, forming 53 of 125 (42.4%) van der Waals contacts and 3 of 8 (37.5%) hydrogen bonds between the TCR and the peptide (Table 4). The HLA helices were contacted by residues within the CDR3α, CDR3β, and CDR2β loops (with additional support of CDR1α residue Tyr^32^), which focused on Arg^65^, Ala^69^, Gln^72^, and Gln^155^ (Fig. 1*C*). HLA residues at positions 65, 69, and 155 are conserved TCR-mediated contact points in several TCR·pHLA-I structures determined so far and are referred to as the “restriction triad” (33).

**TABLE 3:** **Data reduction and refinement statistics (molecular replacement)**

Parameters	PMEL17 TCR·A2-YLE-9V	A2-YLE	A2-YLE-3A	A2-YLE-5A
Protein Data Bank code	5EU6	5EU3	5EU4	5EU5
**Data set statistics**				
Space group	P1	P1 21 1	P1	P1 21 1
Unit cell parameters (Å)	*a* = 45.52, *b* = 54.41, *c* = 112.12, α = 85.0°, β = 81.6°, γ = 72.6°	*a* = 52.81, *b* = 80.37, *c* = 56.06, β = 112.8°	*a* = 56.08, *b* = 57.63, *c* = 79.93, α = 90.0°, β = 89.8°, γ = 63.8°	*a* = 56.33, *b* = 79.64, *c* = 57.74, β = 116.2°
Radiation source	DIAMOND I03	DIAMOND I03	DIAMOND I02	DIAMOND I02
Wavelength (Å)	0.9763	0.9999	0.9763	0.9763
Measured resolution range (Å)	51.87–2.02	45.25–1.97	43.39–2.12	43.42–1.54
Outer Resolution Shell (Å)	2.07–2.02	2.02–1.97	2.18–2.12	1.58–154
Reflections observed	128,191 (8,955)	99,442 (7,056)	99,386 (7,463)	244,577 (17,745)
Unique reflections	64,983 (4,785)	30,103 (2,249)	49,667 (3,636)	67,308 (4,962)
Completeness (%)	97.7 (96.7)	98.5 (99.3)	97.4 (96.7)	99.6 (99.9)
Multiplicity	2.0 (1.9)	3.3 (3.1)	2.0 (2.1)	3.6 (3.6)
I/σ(I)	5.5 (1.9)	7.2 (1.9)	6.7 (2.3)	13 (2.3)
*R*_pim_ (%)	5.7 (39.8)	8.8 (44.7)	8.7 (41.6)	4.5 (35.4)
*R*_merge_ (%)	7.8 (39.6)	9.8 (50.2)	8.7 (41.6)	5.0 (53.2)

**Refinement statistics**				
Resolution (Å)	2.02	1.97	2.12	1.54
No. of reflections used	61,688	28,557	47,153	63,875
No. of reflections in *R*_free_ set	3294	1526	2514	3406
*R*_cryst_ (no cut-off) (%)	18.1	19.7	17.2	17.0
*R*_free_	22.2	25.5	21.1	20.1
Root mean square deviation from ideal geometry				
Bond lengths (Å)	0.018 (0.019)*^a^*	0.019 (0.019)	0.021 (0.019)	0.018 (0.019)
Bond angles (degrees)	1.964 (1.939)	1.961 (1.926)	2.067 (1.927)	1.914 (1.936)
Overall coordinate error (Å)	0.122	0.153	0.147	.055
Ramachandran statistics				
Most favored	791 (96%)	371 (98%)	749 (99%)	384 (98%)
Allowed	32 (4%)	6 (2%)	10 (1%)	5 (1%)
Outliers	2 (0%)	3 (1%)	1 (0%)	2 (0%)

*^a^* Values in parentheses are for the highest resolution shell.

**TABLE 4:** **PMEL17 TCR·A2-YLE-9V contact table**

HLA/peptide residue	TCR residue	No. of van der Waals contacts (≤4 Å)	No. of hydrogen bonds (≤3.4 Å)
Gly^62^	αGly^98^	3	
	αSer^99^	1	
Arg^65^	αSer^99^	2	
Arg65 O	αAsn^100^ Nδ2	2	1
Arg65 NH1	βAsp^58^ Oδ2		1
	βSer^59^	8	
Lys^66^	αGly^98^	1	
	αSer^99^	4	
	αAsn^100^	4	
Ala^69^	αAsn^100^	2	
	βAla^56^	2	
Gln^72^ Nϵ2	βGln^51^ O	3	1
	βGly^54^	7	
	βAla^55^	1	
Thr^73^	βGln^51^	1	
Val^76^	βGln^51^	3	
	βGly^52^	2	
Lys^146^	βPhe^97^	3	
	βIle^98^	3	
Ala^150^	βIle^98^	1	
	βAsp^102^	3	
Val^152^	βIle^98^	1	
Glu^154^	αTyr^32^	1	
Gln^155^ N	αTyr^32^ OH	4	1
Gln^155^ Oϵ1	βThr^101^ N	10	1
Tyr^1^ OH	αGly^97^ O	1	1
	αGly^98^	1	
	αSer^96^	1	
Glu^3^	αTyr^101^	1	
Pro^4^	αSer^96^	1	
	αSer^99^	1	
	αAsn^100^	4	
Pro^4^ O	αTyr^101^ N	14	1
Gly^5^	αTyr^101^	3	
	βGly^100^	2	
Val^7^	βIle^98^	7	
	βGly^99^	2	
	βGly^100^	2	
Thr^8^	βThr^31^	5	
	βGln^51^	1	
	βPhe^97^	1	
Thr^8^ N	βIle^98^ O	6	1

**FIGURE 3.**
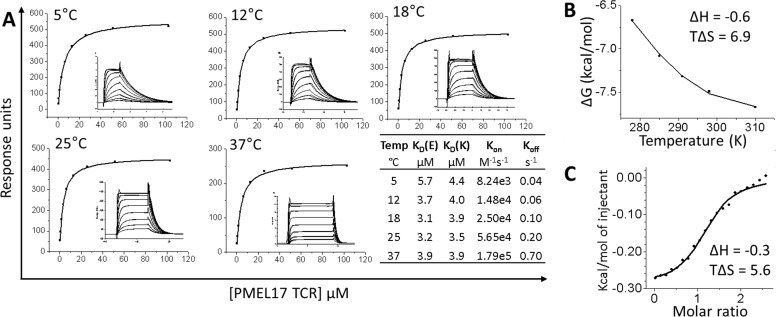
**Thermodynamic analysis of the PMEL17 TCR·A2-YLE interaction.** *A*, PMEL17 TCR equilibrium-binding responses to A2-YLE at 5, 12, 18, 25, and 37 °C across 9–10 TCR serial dilutions. SPR raw and fitted data (assuming 1:1 Langmuir binding) are shown in the *inset* of each *curve* and were used to calculate *K*_on_ and *K*_off_ values using a global fit algorithm (BIAevaluation version 3.1). The *table* shows equilibrium-binding (*K_D_*(*E*)) and kinetic-binding constant (*K_D_*(*K*) = *K*_off_/*K*_on_) at each temperature. The equilibrium binding constant (*K_D_*, μm) values were calculated using a nonlinear fit (*y* = (P_1_*x*)/(P_2_ + *x*)). *B*, the thermodynamic parameters were calculated according to the Gibbs-Helmholtz equation (Δ*G*^0^ = Δ*H* − *T*Δ*S*^0^). The binding free energies, Δ*G*^0^ (Δ*G*^0^ = −*RT*ln*K_D_*), were plotted against temperature (*K*) using nonlinear regression to fit the three-parameter equation (*y* = d*H* + dCp*(*x* − 298) − x*d*S* − x*dCp*ln(*x*/298)). Enthalpy (Δ*H*^0^) and entropy (*T*Δ*S*^0^) at 298 K (25 °C) are shown in kcal/mol and were calculated by a non-linear regression of temperature (K) plotted against the free energy (Δ*G*^0^). *C*, ITC measurements for PMEL17 TCR·A2-YLE interaction. Enthalpy (Δ*H*^0^) and entropy (*T*Δ*S*^0^) at 298 K (25 °C) are shown in kcal/mol.

To complement information gained from the crystal structure, we studied the affinity and thermodynamic parameters of the PMEL17 TCR·A2-YLE complex. The binding strength of the complex was measured at 5, 12, 18, 25, and 37 °C by SPR (Fig. 3*A*). The PMEL17 TCR·A2-YLE interaction at 25 °C (the standard temperature for TCR·pHLA parameter measurements) was characterized by a binding Δ*G* of −7.5 kcal/mol, which is within the normal range of TCR·pHLA interactions (34). PMEL17 TCR·A2-YLE binding was characterized by a very small, favorable enthalpy change (Δ*H* = −0.6 kcal/mol) and a larger, positive entropy change (*T*Δ*S* = 6.9 cal/mol) (Fig. 3*B*). Therefore, order to disorder drove the interaction, probably through the expulsion of ordered water molecules at the interface (*i.e.* solvation effects). ITC was also performed because it provides a direct measure of enthalpy and is therefore considered the most reliable determination of thermodynamic parameters (29). ITC measurements (Δ*H* = −0.3 kcal/mol and *T*Δ*S* = 5.6 cal/mol) confirmed observations made with SPR thermodynamics (Fig. 3*C*). The favorable enthalpy of this TCR·pHLA system shows that the overall number of formed bonds is equal to the number of disrupted ones upon PMEL17 TCR binding.

##### The PMEL17 CDR Loops Focus on Peptide Residues Pro^4^, Val^7^, and Thr^8^

**FIGURE 4.**
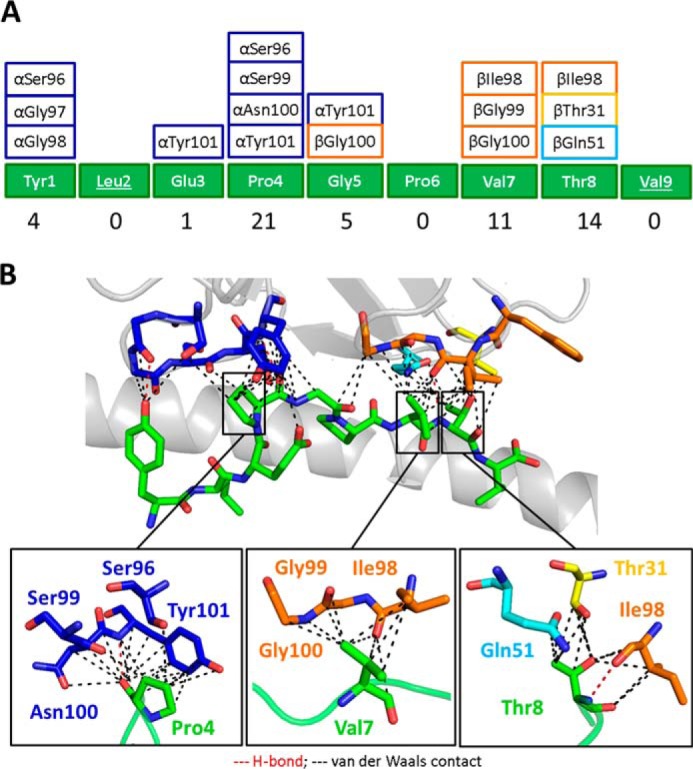
**The PMEL17 CDR loops focus on peptide residues Pro^4^, Val^7^, and Thr^8^.** *A*, schematic representation of contacts between YLE-9V peptide and PMEL17 CDR loop residues (*color-coded* as in Fig. 2*A*). The *numbers* at the *bottom* show total contacts between the TCR and peptide. *B*, contacts between the PMEL17 TCR and the YLE-9V peptide (*green sticks*), showing the van der Waals contacts (*black dashed lines*) and hydrogen bonds (*red dashed lines*) made by the TCR CDR3α (*blue*), CDR1β (*yellow*), CDR2β (*aqua*), and CDR3β (*orange*) loops. *Bottom panel*, close view of contacts between YLE Pro^4^, Val^7^, and Thr^8^, respectively, and TCR CDR loop residues (*sticks color-coded* as in Fig. 1*A*) (cut-off of 3.4 Å for hydrogen bonds and a cut-off of 4 Å for van der Waals contacts).

The central positioning of the PMEL17 TCR enabled contacts with 6 of 9 amino acids in the peptide (Fig. 4*A*). Peptide residues Pro^4^, Val^7^, and Thr^8^ represented the focal points of the PMEL17 TCR. Pro^4^ made a sizeable network of interactions (1 hydrogen bond and 14 van der Waal contacts) (Fig. 4*B*). Interestingly, Pro^6^ was the only central residue that did not interact with the PMEL17 TCR because of its reduced surface exposure. Therefore, the relative insensitivity of the PMEL17 TCR to alanine substitution at position 6 is consistent with its lack of involvement in TCR binding. In contrast, the gp100 TCR seemed to be more sensitive to this same mutation, causing a ∼40-fold drop in binding affinity compared with the unaltered peptide (Table 2). This might be explained by the different *TRAV* and *TRBV* gene usage of the two gp100-specific TCRs and the very different residues of the CDR3 loops possibly contacting the central part of the gp100^280–288^ peptide. However, the PMEL17 TCR complex structure did not provide any clear mechanisms to explain the reduction in binding observed when peptide residues 3 and 5 were mutated to alanine.

##### Peptide Substitutions Can Induce Perturbation at Adjacent Peptide Residues Abrogating T-cell Recognition

**FIGURE 5.**
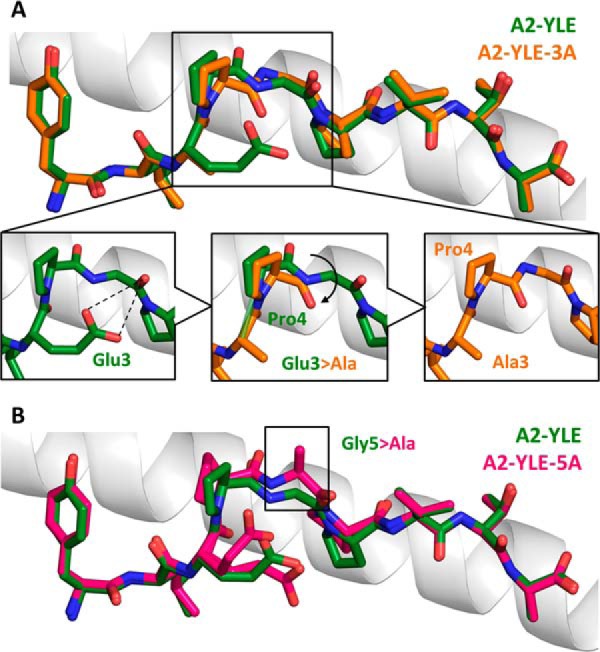
Conformational comparison of YLE, YLE-3A, and A2-YLE-5A peptides presented by HLA-A*0201. *A*, YLE (*dark green sticks*) and YLE-3A (*orange sticks*) peptide alignment by superimposition of HLA-A*0201 α1 helix (*gray schematic*). *Boxed residues* indicate the mutation of Glu^3^ into an alanine. The *insets* show how the Glu^3^ → Ala substitution causes a shift in position (*black arrow*) of neighbor residue Pro^4^ in the A2-YLE-3A structure compared with the A2-YLE structure. *B*, YLE (*dark green sticks*) and YLE-5A (*pink sticks*) peptide alignment by superimposition of HLA-A*0201 α1 helix (*gray schematic*). *Boxed residues* indicate the mutation of glycine 5 into an alanine.

In order to understand the structural basis underlying the large changes in affinity of PMEL17 TCR·A2-YLE binding resulting from Glu^3^ → Ala and Gly^5^ → Ala substitutions, we also solved the unligated structures of A2-YLE, A2-YLE-3A, and A2-YLE-5A. The structures were solved between 1.54 and 2.12 Å resolution with crystallographic *R*_work_/*R*_free_ ratios within accepted limits (Table 3) (31). Electron density around the peptide was unambiguous (Fig. 1). Comparison of the crystallographic structure of A2-YLE and A2-YLE-3A or A2-YLE-5A complexes did not reveal major changes in the peptide backbone conformation (Fig. 5, *A* and *B*). However, in the A2-YLE structure, Glu^3^ bridges across to the main chain at position 4–5; the mutation of Glu^3^ into a shorter alanine side chain, which is no longer able to bridge across the void, caused a knock-on effect on the central Pro^4^ residue (Fig. 5*A*). This difference could not be explained by the difference in resolution and coordinate errors in the A2-YLE-3A structure (A2-YLE-3A was solved at 2.12 Å, compared with 1.97 Å for A2-YLE and 1.54 Å for A2-YLE-5A), demonstrated by omit map analysis shown in Fig. 1. Pro^4^ in the A2-YLE-3A structure lost restraint, causing the oxygen atom to flip in the opposite direction. Because of this unanticipated displacement of the Pro^4^ oxygen, the outward facing side of the Pro^4^ residue was no longer in an optimal position to be contacted by the TCR, therefore potentially losing the dominant network of interactions (Fig. 4*B*). These findings explain the complete absence of measurable binding of the YLE-3A mutant in the alanine scan. Gly^5^ was the only gp100^280–288^ peptide residue contacted by both CDR3 loops through αTyr^101^ and βGly^100^ in the PMEL17 TCR·A2-YLE-9V structure (Fig. 5*B*). In the A2-YLE-5A structure, steric hindrance in the center of the peptide may explain the substantial reduction in binding affinity observed in the alanine scan. As with YLE-3A, the YLE-5A substitution did not distort the overall conformation of the YLE nonamer.

##### Alanine Substitutions at YLE Peptide Residues 3 and 5 Abrogate T-cell Activation

**FIGURE 6.**
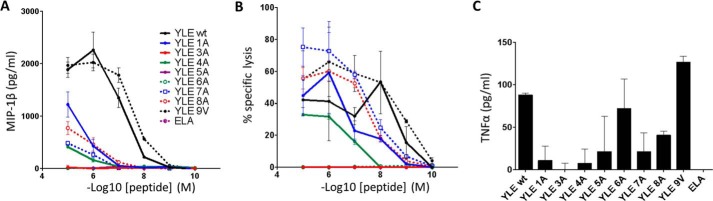
**Production of MIP-1β and TNFα and cytotoxicity by gp100 TCR-transduced CD8^+^ T-cells in response to stimulation with YLE peptide ligands.** *A*, gp100 TCR-transduced CD8^+^ T-cells were stimulated with peptide-pulsed target cells as indicated. Supernatant was assayed for MIP-1β by ELISA. *B*, gp100 TCR-transduced CD8^+^ T-cells were tested in a standard 4-h ^51^Cr release assay against peptide-pulsed targets. *C*, gp100 TCR-transduced CD8^+^ T-cells were stimulated with peptide-pulsed target cells (10^−5^ m peptide) as indicated. Supernatant was assayed for TNFα by ELISA. ELA (Melan-A/MART-1) peptide was used as an irrelevant control in all experiments. Results of one donors (of three) are shown. *Error bars*, S.E.

To determine the effect of gp100^280–288^ altered peptide ligands on the activation of T-cells, we examined the ability of these mutants to induce MIP-1β, TNFα production and specific cytotoxicity (Fig. 6). These are key effector functions of activated CD8^+^ T-cells, which can be measured over a wide range of peptide concentrations. Human primary CD8^+^ T-cells were transduced with a lentiviral construct carrying the gp100 TCR and enriched for high and equal levels of TCR expression. Staining with TCRVβ mAb showed that ∼40% of total CD8^+^ T-cells stained as positive for gp100 TCR expression by flow cytometry in three independent donors (data not shown). Transduced CD8^+^ T-cells were stimulated with peptide-pulsed HLA-A*0201^+^ T2 target cells, across a range of different concentrations of gp100^280–288^ altered peptide ligands. Antigen-specific responses of gp100 TCR-engineered T-cells were validated at the level of production of MIP-1β and specific lysis of pulsed targets. Non-transduced CD8^+^ T-cells were used to control for nonspecific activation through the endogenous TCR; T-cells transduced with the MEL5 TCR (specific for the HLA-A*0201 restricted cancer epitope ELA from the Melan-A/MART-1 protein) were used as an irrelevant control in all experiments (data not shown). Peptide titration experiments showed marked differences in the ability to sensitize target cells for MIP-1β production by CD8^+^ gp100-specific T-cells (Fig. 6*A*). In particular, target cells pulsed with YLE and YLE-9V were recognized more efficiently than those pulsed with YLE-1A, YLE-8A, YLE-4A, and YLE-7A. No MIP-1β production was measured with YLE-3A and YLE-5A peptide ligands, even at higher peptide concentrations. TNFα production was measured by ELISA from the same supernatants (Fig. 6*C*), and low levels of this cytokine were only detected when cells were pulsed with YLE, YLE-9V, YLE-8A, or YLE-6A. Fig. 6*B* shows specific lysis of target cells pulsed with the same range of peptides and measured by a ^51^Cr release assay. Similar to the MIP-1β response curves, the specific lysis induced by these altered gp100^280–288^ ligands was variable. Most importantly, no cytotoxic T lymphocyte-mediated lysis was observed when peptide YLE-3A or YLE-5A was used. Taken together, these data are consistent with the molecular analysis demonstrating that the structural and biophysical alterations induced by peptide modifications translate directly to the effects that we observed upon T-cell recognition.

## Discussion

TCRs specific for cancer epitopes are generally characterized by low binding affinities (binding *K_D_* values in the high micromolar range) (35). This lower binding affinity is thought to be a result of negative selection of T-cells that bear TCRs with higher affinity for self-ligands in the thymus. Because TCR affinity plays an important role in T-cell activation, the TCR affinity gap between anti-pathogen and anti-cancer T-cells leaves the latter at a distinct disadvantage and makes it more difficult to break self-tolerance to such antigens. One approach to enhance the T-cell response to tumor antigen-derived peptides has been to immunize patients with altered peptide ligands that differ from the native sequence by a single or multiple amino acid residues. However, such “heteroclitic” peptides with even single amino acid substitutions that are predicted to only contact the HLA can have unpredictable, yet important, effects on TCR engagement. To date, only a few x-ray structures of TCRs bound to cognate tumor antigens have been determined (18, 36–38). Given the growing evidence that plasticity at the TCR·pHLA interface can influence immune recognition (39), structural and biophysical studies should be taken into account when attempting to design altered peptide ligands with improved immunogenicity.

We solved the first structure of a naturally occurring αβTCR in complex with a gp100 HLA-A*0201-restricted melanoma epitope. Overall, the PMEL17 TCR bound with a typical diagonal orientation over the central peptide residues and mainly contacted residues 4, 7, and 8 of the YLE peptide, which protruded out of the HLA-A*0201 binding groove. It is important to underscore that the PMEL17 TCR was characterized by a binding affinity (*K_D_*) of 7.6 μm. This value falls at the very high end of the affinity range described so far for cancer TCRs (11, 35). These results suggest that T-cells bearing TCRs with reasonable affinity for some tumor-associated antigens may escape central tolerance, opening the door to further TCR engineering for medical applications (40).

We also provide insight into the role of each residue in gp100^280–288^ during TCR recognition by performing an alanine scan mutagenesis with two different gp100^280–288^-specific αβTCRs. With regard to HLA anchor-modified “heteroclitic” peptides, previous studies have shown that even highly immunogenic designer peptides (e.g. ELA epitope from Melan-A/MART-1 protein) do not necessarily induce a better clinical response (13, 41, 42). Fortunately, this is not the case for the gp100 YLE-9V peptide, which has been successfully adopted in clinical trials (15). These observations are consistent with our *in vitro* findings, in that the A2-YLE-9V bound with similar affinity to PMEL17 TCR and gp100 TCR compared with the native peptide. Interestingly, both the PMEL17 TCR (*TRAV21 TRBV7-3*) and gp100 TCR (*TRAV17 TRBV19*) were most sensitive to mutations at position 3 or 5 of the native gp100^280–288^ peptide sequence despite these TCRs being constructed from completely different Vα and Vβ genes. A previous study of gp100 altered peptide ligands also showed the YLE-3A mutant to be a null agonist when tested on gp100-specific TCR-transfected human T-cells (17). Our results provide a molecular explanation for this finding.

We show that PMEL17 TCR non-responsiveness to A2-YLE-3A was caused by an unexpected molecular switch in the peptide, repositioning the Pro^4^ residue, which was at the center of a sizeable network of interactions (both van der Waals contacts and hydrogen bonds) in the PMEL17·A2-YLE-9V structure. Position 3 in HLA-A*0201 restricted peptides is known to be a secondary anchor residue (43), in that it supports the exposed peptide bulge that is normally involved in TCR binding. Interestingly, mutation in position 3 in the YLE peptide did not alter the conformation of the peptide backbone itself but resulted in a “knock-on” effect on the neighboring residue Pro^4^ that completely abolished TCR binding and T-cell recognition. We have recently described a similar molecular switch in an HIV-1-derived peptide, with important implications for the immune control of HIV infection and patterns of viral escape mutants (44). Additionally, we have demonstrated the existence of a novel mode of flexible peptide presentation in a diabetes model, showing the dynamic nature of the region surrounding the HLA F-pocket (39, 45). Taken together, these studies highlight that the peptide-HLA interaction is more plastic and dynamic than previously appreciated, with obvious implications for immune recognition, epitope prediction, and structural modeling.

Overall, our results represent the first structural insight into TCR recognition of an important tumor antigen, targeted by many clinical therapies. These data reveal that two very different TCRs share a similar pattern of specificity, demonstrated by their nearly identical sensitivity to different peptide modifications. Finally, we show that even changes in a single peptide residue that are not heavily engaged by a TCR can have important, knock-on effects on other residues in an HLA-bound peptide that can dramatically alter T-cell recognition. Such “transmitted” structural changes need to be taken into consideration when designing improved peptides for cancer vaccination.

## Author Contributions

V. B., G. D., A. B., G. D., A. F., A. T., P. J. R., and D. K. C. performed experiments and analyzed the data. V. B., A. K. S., and D. K. C. wrote the manuscript. A. K. S. and D. K. C. conceived and directed the study. A. K. S. and D. K. C. funded the study. All authors contributed to discussions.

## Footnotes

6 The abbreviations used are:

pHLA: peptide human leukocyte antigen
TCR: T-cell receptor
gp100: glycoprotein 100
SPR: surface plasmon resonance
Bistris propane: 1,3-bis[tris(hydroxymethyl)methylamino]propane
ITC: isothermal titration calorimetry.
